# Dental education changed by COVID-19: Student’s perceptions and attitudes

**DOI:** 10.1186/s12909-021-02806-5

**Published:** 2021-07-03

**Authors:** Hsin-Chung Cheng, Sao-Lun Lu, Yu-Chun Yen, Pallop Siewchaisakul, Amy Ming-Fang Yen, Sam Li-Sheng Chen

**Affiliations:** 1 School of Dentistry, College of Oral Medicine, Taipei Medical University, No.250, Wuxing St., Xinyi Dist, Taipei, 110 Taiwan; 2 Division of Orthodontics, Department of Dentistry, Taipei Medical University Hospital, Taipei, Taiwan; 3 Research Center of Biostatistics, College of Management, Taipei Medical University, Taipei, Taiwan; 4 Faculty of Public Health, Chiang Mai University, Chiang Mai, Thailand; 5 School of Oral Hygiene, College of Oral Medicine, Taipei Medical University, No.250, Wuxing St., Xinyi Dist, Taipei, 110 Taiwan

**Keywords:** Dental education, Infection control, COVID-19, Online education, Attitudes, Perceptions

## Abstract

**Background:**

Dental students have encountered changes in the teaching format amid the SARS CoV-2 pandemic. This study aims to evaluate the attitudes of dental students of one medical university toward online courses and compare them with those of non-dental students amid the SARS CoV-2 pandemic.

**Methods:**

A cross-sectional survey with a self-report online questionnaire was conducted at the medical university in May 2020 in Taipei. Students from the School of Dentistry, School of Dental Technology, and School of Oral Hygiene Study were enrolled in our survey.

**Results:**

In total, 473 students responded to the survey, 318 (67.2%) of whom were dental students. Overall, 366 (77%) students agreed with the change to online learning. Only 10.4% of students thought that dental professional courses with a laboratory format could be changed to online courses. Dental students were significantly more worried than non-dental students about being infected with COVID-19 and about the COVID-19 pandemic continuing.

**Conclusions:**

In conclusion, changing to online learning seems to be perceived as feasible by students. However, more discussion about changing dental professional courses with a laboratory format to online courses considering the attitudes from students is needed.

**Supplementary Information:**

The online version contains supplementary material available at 10.1186/s12909-021-02806-5.

## Introduction

Coronavirus disease 2019 (COVID-19) has been noted worldwide as an infectious disease caused by severe acute respiratory syndrome coronavirus 2 (SARS CoV-2). Several containment measures have been applied to stop the pandemic contagion of the virus, including isolation, contact tracing and quarantine, physical distancing, hygiene measures and lockdown [1, 2]. In particular, one measure, so-called social distancing or physical distancing, has been widely used. The main goal of social distancing is to protect ourselves and others from the spread of COVID-19 [3].

Inevitably, education is one of the fields that has required social distancing. Experience from influenza outbreaks has shown that school closure can interrupt virus transmission because it reduces social contact between students [4]. The UN Educational, Scientific and Cultural Organization estimated that approximately 107 countries had conducted national school closures as of March 18, 2020. These closures affected approximately half of the student population worldwide [5].

Dental education in both dental clinics and preclinical laboratories has been affected by the virus [6]. Recently, traditional face-to-face classroom educational activities with undergraduate and postgraduate dentistry students were found to be interrupted nearly worldwide [7].

Dental professionals are at higher risk of becoming infected with the virus, especially when patients are asymptomatic or mildly symptomatic, because dental practice involves face-to-face communication with patients and frequent exposure to saliva, blood, and other body fluids [8]. Moreover, routine dental clinical procedures that generate droplets and aerosols are thought to be one of the modes of transmission to patients, dentists, and related staff [9]. This situation was also reported in Taiwan [10].

As a result, many faculty dentistry around the world have rapidly adopted virtual modes. Staff and professors have provided high-quality remote courses from their homes [11–13]. Although online courses are considered to provide safety benefits for students, students’ perceptions of and attitudes toward online courses might not be as good as expected [14]. Furthermore, to control the spread of the virus, knowledge and awareness of the disease should be increased. However, previous studies revealed that dental students, residents, and specialists lacked knowledge and awareness of viral infections in relation to human immunodeficiency virus (HIV) and hepatitis-B virus (HBV) [15].

To our knowledge, very few studies have examined the attitudes of dental students toward changes in dental education and COVID-19. Knowing these perceptions of and attitudes during the spread of the virus would also help policy makers and authorized university staff to plan and manage dental education and control the outbreak.

We therefore aim to evaluate the attitudes of dental students toward online courses and compare them with those of non-dental students who are associated with the School of Dentistry at a medical university amid the SARS CoV-2 pandemic.

## Materials and methods

### Study sample

During the COVID-19 pandemic, a cross-sectional study was conducted at College of Oral Medicine in a medical university in May 2020. In Taiwan, undergraduate programme of School of Dentistry (SD) requires students to complete 1 to 6 study years, and progrmmes of School of Dental Technology (SDT) and School of Oral Hygiene (SOH) require students to complete 1 to 4 study years. Students from SD are required to complete the internship in the sixth year. Students from SDT and SOH are required to attend the onsite course in the practice field in the fourth year. To avoid the disturbance of internship and field practice, we included only students from the first to the fourth year of SD and the first to the third year students from the SDT and SOH to this survey. The respondents were further divided into dental (SD) and non-dental (STD and SOH) students. The questionnaire was delivered via an e-Learning online system. The completed questionnaire was returned via the same online system. The questionnaire return rate was 70% (473/674). There were no strict selection criteria for the participating students. The study was approved by the Institutional Review Board (TMU-JIRB: N202004124).

### Changes in teaching and learning during the COVID-19 pandemic

To control and prevent the pandemic COVID-19, all universities in Taiwan postponed school reopening until March 2, 2020, following the announcement from the Ministry of Education, Taiwan. In early February, the study university established a COVID-19 prevention working group to act as the operational command for the COVID-19 response on campus. The working group asked all students and faculty members on campus to wear masks, maintain social distancing, and provide good environmental controls in the classroom. Classes with over 100 students were required to shift to online courses. Other courses with a small number of students were also suggested to be changed to online courses. Most of the faculty members of the university understood the need and importance of online courses in such a situation, and all understood they should adopt technology change. With the crisis, there was a wide adoption of technology for both teaching and learning. The tools used for online courses during the epidemic period for teaching and learning included PowerPoint lecture recorders, online education platforms, video meeting applications, etc.

### Questionnaire

The survey instrument used was a self-administered questionnaire. The anonymous questionnaire contained 29 questions in three major categories: demographic characteristics (total of 3 questions); perceptions of and attitudes toward online courses (total of 12 questions); and perceptions of and attitudes toward the impact of COVID-19 (total of 14 questions). The students can choose only one of the five level of an agreement, including strongly agree, agree, neutral, disagree and strongly disagree. All questions in the questionnaire were designed by two experts. The questionnaire was reviewed and validated the contents by three of the expert reviewers. The experts conducted their review independently.

### Statistical analysis

Categorical variables were summarized as counts (percentages). The comparisons of the attitudes of dental and non-dental students toward online courses and COVID-19 were performed using the chi-square test. All statistical analyses were performed with SAS 9.4.

## Results

Table 1 shows the demographic data on gender and grade for dental and non-dental students. There was a significant difference in both gender and grade between the two groups. Most of the students in the dental student group were male 181 (56.92%), whereas most of the students in the non-dental student group were female 117 (75.48%). Regarding grade, the highest grade among non-dental students was grade 3, with the largest proportion of non-dental students in this grade 57 (36.77%). However, we found that most dental students were in the first grade 76 (23.90%).

**Table 1:** Demographic distribution of dental and non-dental students

	Dental student, *n* = 318		Non-dental student, *n* = 155		
Grade/Gender	n	%	n	%	*P*-value
Grade					**< 0.0001**
1	76	23.90	51	32.90	
2	67	21.07	47	30.32	
3	53	16.67	57	36.77	
4	71	22.33			
5	51	16.04			
Gender					**< 0.0001**
Male	181	56.92	38	24.52	
Female	137	43.08	117	75.48	

Table 2 presents students’ attitudes toward online learning. Many students strongly agreed with item 1–1, “I agree with changing on-site courses to online courses” 190 (40.17%), and item 1–6, “I think other non-medical professional courses with a lecture format could be changed to online courses” 202 (42.71%).

**Table 2:** Attitudes toward online courses among dental and non-dental students

Questions	Strongly Agree		Agree		Neutral		Disagree		Strongly disagree	
	n	%	n	%	n	%	n	%	n	%
1–1. I agree with changing on-site courses to online courses	190	**40.17**	176	37.21	88	18.60	13	2.75	6	1.27
1–2. Online courses change my learning schedule and behavior	45	9.51	282	**59.62**	120	25.37	18	3.81	8	1.69
1–3. Online courses change my way of learning	69	14.59	202	**42.71**	157	33.19	40	8.46	5	1.06
1–4. I am satisfied with the learning efficacy of online courses	142	30.02	79	16.70	196	**41.44**	46	9.73	10	2.11
1–5. The learning efficacy of online courses is better than that of on-site courses	86	18.18	111	23.47	174	**36.79**	75	15.86	27	5.71
1–6. I think other non-medical professional courses with a lecture format could be changed to online courses	202	**42.71**	173	36.58	65	13.74	25	5.29	8	1.69
1–7. I think other non-medical professional courses with a lecture format could be changed to online courses	116	24.52	151	**31.92**	123	26.00	55	11.63	28	5.92
1–8. I think dental professional courses with a laboratory format could be changed to online courses	19	4.02	30	6.34	66	13.95	140	29.60	218	46.09
	1 Month		3 Months		6 Months		9 Months		12 Months	
	n	%	n	%	n	%	n	%	n	%
1–9. I could tolerate the learning style of an online course for …	62	13.11	134	28.33	94	19.87	13	2.75	170	35.94

A large proportion of students agreed with items 1–2, “Online courses change my learning schedule and behavior”, 1–3, “Online courses change my way of learning”, and 1–7, “I think dental professional courses with a lecture format could be changed to online courses”, with 282 (59.62%), 202 (42.71%) and 151 (31.92%) of students, respectively, agreeing with these questions. Items 1–4, “I am satisfied with the learning efficacy of online courses”, and 1–5, “The learning efficacy of online courses is better than that of on-site courses” received “neutral” responses from 196 (41.44%) and 174 (36.79%) of students, respectively. Approximately one-third of students thought they could continue learning via online courses for a year.

Students’ attitudes toward COVID-19 and education are shown in Table 3. Neutral responses were the most common responses for the following questions: 2–1, “Are you pessimistic about the development of COVID-19?” 202 (42.71%); 2–4, “Would you actively gather the latest medical information about COVID-19 and in-depth knowledge?” 218 (46.09%); 2–5, “Has COVID-19 affected your original plans for choosing a career in the future?” 196 (41.44%); 2–9, “Has COVID-19 changed your daily schedule and habits?” 195 (41.23%); 2–11: “Are you worried about being infected with COVID-19?” 235 (49.68%), 2–12: “Are you worried that COVID-19 will continue?” 171 (36.15%); 2–13, “Are you worried that the epidemic will affect your learning?” 186 (39.32%); 2–15, “Has your learning method changed based on the current online learning mode?” 208 (43.97%); 2–16, “How satisfied are you with your self-learning outcomes with the current online learning mode?” 196 (41.44%); and 2–17: “Do you think the current online learning is more effective than physical courses?” 179 (37.84%).

**Table 3:** Attitudes toward COVID-19 among dental and non-dental students

	Strongly Agree		Agree		Neutral		Disagree		Strongly Disagree	
	n	%	n	%	n	%	n	%	n	%
Mental Health										
2–1. Are you pessimistic about the development of COVID-19?	11	2.33	70	14.8	202	**42.71**	143	30.23	47	9.94
2–2. Do you feel worried and sleepless when you think about COVID-19?	3	0.63	14	2.96	92	19.45	186	**39.32**	178	37.63
Knowledge										
2–3. Would you actively seek out and be concerned about the news about and development of COVID-19 at home and abroad?	92	19.45	232	**49.05**	128	27.06	19	4.02	2	0.42
2–4. Would you actively gather the latest medical information about COVID-19 and in-depth knowledge?	46	9.73	165	34.88	218	**46.09**	39	8.25	5	1.06
Social Network and Behavior										
2–5. Has COVID-19 affected your original plans for choosing a career in the future?	10	2.11	43	9.09	196	**41.44**	160	33.83	64	13.53
2–6. Has COVID-19 changed your social interaction?	58	12.26	189	**39.96**	147	31.08	63	13.32	16	3.38
2–7. Has COVID-19 changed your way of relaxing?	102	21.56	206	**43.55**	116	24.52	38	8.03	11	2.33
2–8. Has COVID-19 changed your personal hygiene?	122	25.79	211	**44.61**	103	21.78	27	5.71	10	2.11
2–9. Has COVID-19 changed your daily schedule and habits?	47	9.94	141	29.81	195	**41.23**	71	15.01	19	4.02
2–10. Has COVID-19 changed your learning mode?	69	14.59	220	**46.51**	132	27.91	38	8.03	14	2.96
Attitudes										
2–11. Are you worried about being infected with COVID-19?	10	2.11	62	13.11	235	**49.68**	111	23.47	55	11.63
2–12. Are you worried that COVID-19 will continue?	7	1.48	51	10.78	171	**36.15**	167	35.31	77	16.28
2–13. Are you worried that the epidemic will affect your learning?	26	5.50	97	20.51	186	**39.32**	117	24.74	47	9.94
2–14. Are you worried that COVID-19 create financial pressure for your school studies?	38	8.03	170	**35.94**	145	30.66	78	16.49	42	8.88
Efficacy of Online Courses										
2–15. Has your learning method changed based on the current online learning mode?	36	7.61	121	25.58	208	**43.97**	86	18.18	22	4.65
2–16. How satisfied are you with your self-learning outcomes with the current online learning mode?	32	6.77	115	24.31	196	**41.44**	102	21.56	28	5.92
2–17. Do you think the current online learning is more effective than physical courses?	31	6.55	127	26.85	179	**37.84**	97	20.51	39	8.25

A total of 232 (49.05%), 189 (39.96%), 206 (43.55%), 211 (44.61%), 220 (46.51%) and 170 (35.94%) of students agreed with question 2–3, “Would you actively seek out and be concerned about the news about and development of COVID-19 at home and abroad?”; question 2–6, “Has COVID-19 changed your social interaction?”; question 2–7, “Has COVID-19 changed your way of relaxing?”; question 2–8, “Has COVID-19 changed your personal hygiene?”; question 2–10, “Has COVID-19 changed your learning mode?”; and question 2–14, “Are you worried that COVID-19 will create financial pressure for your school studies?”, respectively. More than two-thirds of the students disagreed or strongly disagreed with question 2–2, “Do you feel worried and sleepless when you think about COVID-19?”

Table 4 shows the comparison of the responses of dental students and non-dental students to the questions reported in the previous paragraphs. We found 8 items with statistically significant differences in “agree” responses between the two groups, including 1–7, “I think other non-medical professional courses with a lecture format could be changed to online courses”; 1–8, “I think dental professional courses with a laboratory format could be changed to online courses”; 2–1, “Are you pessimistic about the development of COVID-19?”; 2–2, “Do you feel worried and sleepless when you think about COVID-19?”; 2–5, “Has COVID-19 affected your original plans for choosing a career in the future?”; 2–11, “Are you worried about being infected with COVID-19?”; 2–12, “Are you worried that COVID-19 will continue?”; and 2–14, “Are you worried that COVID-19 will create financial pressure for your school studies?”

**Table 4:** Responses (neutral, agree, and strongly agree) of dental and non-dental students regarding their attitudes toward online courses and COVID-19

Questions	Dental students *n* = 318		Non-dental students *n* = 155		
	n	%	n	%	*P*-value
1–1. I agree with changing on-site courses to online courses	304	95.60	150	96.77	0.54
1–2. Online courses change my learning schedule and behavior	297	93.40	150	96.77	0.13
1–3. Online courses change my way of learning	282	88.68	146	94.19	0.06
1–4. I am satisfied with the learning efficacy of online courses	281	88.36	136	87.74	0.84
1–5. The learning efficacy of online courses is better than that of on-site courses	248	77.99	123	79.35	0.73
1–6. I think other non-medical professional courses with a lecture format could be changed to online courses	293	92.14	147	94.84	0.28
1–7. I think dental professional courses with a lecture format could be changed to online courses	253	79.56	137	**88.39**	**0.018**
1–8. I think dental professional courses with a laboratory format could be changed to online courses	59	18.55	56	**36.13**	**<.0001**
2–1. Are you pessimistic about the development of COVID-19?	180	56.60	103	**66.45**	**0.040**
2–2. Do you feel worried and sleepless when you think about COVID-19?	63	19.81	46	**29.68**	**0.017**
2–3. Would you actively seek out and be concerned about the news about and development of COVID-19 at home and abroad?	304	95.60	148	95.48	0.96
2–4. Would you actively gather the latest medical information about COVID-19 and in-depth knowledge?	285	89.62	144	92.90	0.25
2–5. Has COVID-19 affected your original plans for choosing a career in the future?	149	46.86	100	**64.52**	**0.0003**
2–6. Has COVID-19 changed your social interaction?	267	83.96	127	81.94	0.58
2–7. Has COVID-19 changed your way of relaxing?	284	89.31	140	90.32	0.73
2–8. Has COVID-19 changed your personal hygiene?	295	92.77	141	90.97	0.49
2–9. Has COVID-19 changed your daily schedule and habits?	262	82.39	121	78.06	0.26
2–10. Has COVID-19 changed your learning mode?	278	87.42	143	92.26	0.11
2–11. Are you worried about being infected with COVID-19?	222	**69.81**	85	54.84	**0.001**
2–12. Are you worried that COVID-19 will continue?	174	**54.72**	55	35.48	**<.0001**
2–13. Are you worried that the epidemic will affect your learning?	213	66.98	96	61.94	0.28
2–14. Are you worried that COVID-19 will create financial pressure for your school studies?	247	**77.67**	106	68.39	**0.029**
2–15. Has your learning method changed based on the current online learning mode?	247	77.67	118	76.13	0.71
2–16. How satisfied are you with your self-learning outcomes with the current online learning mode?	231	72.64	112	72.26	0.93
2–17. Do you think the current online learning is more effective than physical courses?	228	71.70	109	70.32	0.76

**Fig. 1:**
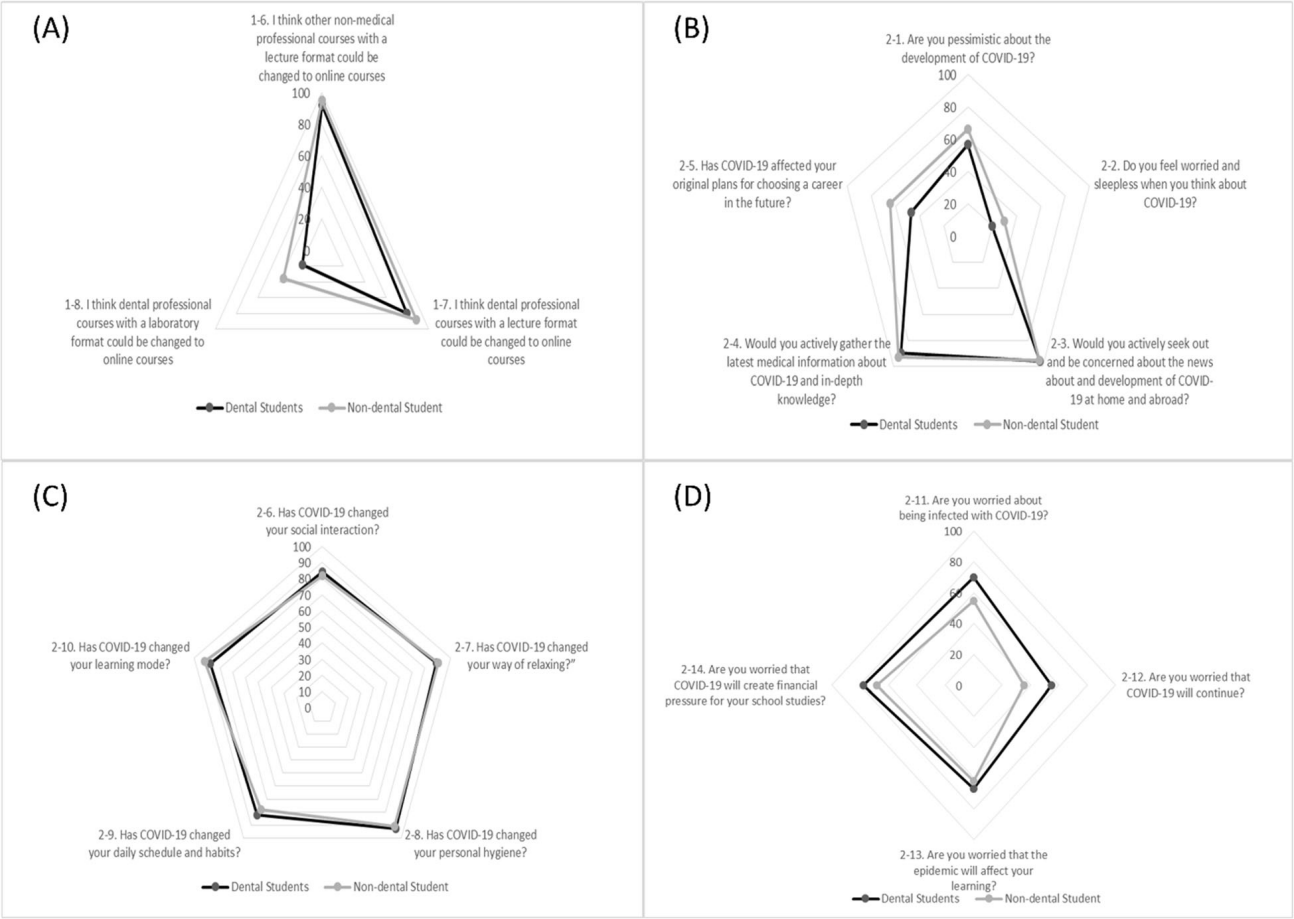
**A** Attitudes toward changing from traditional to online courses. **B** & **C** Attitudes regarding the impact of COVID-19 on students’ lives. **D** Worries about COVID-19

The responses of dental and non-dental students are also shown for groups of items as radar charts in Figs. 1. Figure 1 (A) indicates that dental students agreed less than non-dental students that dental professional courses with both laboratory and lecture formats could be changed to online courses. Dental students also seemed to be less worried, to be less likely to feel pessimistic about COVID-19 and to be less likely to think that the COVID-19 pandemic had affected their original plans for choosing their future careers than non-dental students; however, both student groups highly agreed that they actively updated themselves on information about COVID-19 (Fig. 1(B)).

Fig. 1 (C): Both student groups strongly agreed that COVID-19 changed their ways of relaxing, daily schedules and habits, personal hygiene, and social interaction and learning modes. As demonstrated in Fig. 1(D), dental students were more worried about COVID-19 affecting their learning, creating financial pressure for their school studies, and being infected with COVID-19 and were more worried that the virus would continue

## Discussion

Due to the crisis of the SARS CoV-2 pandemic, traditional face-to-face classes have been changed to virtual online classes in dental education. However, information on students’ attitudes toward online courses during the COVID-19 pandemic is still limited. We evaluated the attitudes of dental students at a medical university toward online courses and compared their attitudes with those of non-dental students at the same medical university.

We found that most students had positive attitudes toward changing on-site learning to online learning and thought that online courses were better than on-site courses. However, they were neutral regarding the efficacy of online courses. They thought that changing to online courses is feasible for non-dental and dental professional courses but not for dental professional courses with laboratory formats.

Regarding COVID-19, the surveyed students were neutral regarding worries about the COVID-19 pandemic and the virus affecting their original plans for choosing career. However, they strongly agreed that COVID-19 affected their social interaction, financial pressure and ways of life.

We further compared the responses of dental and non-dental students and found that non-dental students were more likely than dental students to agree that dental professional courses with both laboratory and lecture formats could be changed to online courses. The non-dental students were significantly more likely than dental students to report worrying, being pessimistic about COVID-19 and thinking that the virus had affected their original plans for choosing their future careers. However, dental students were significantly more worried about being infected with COVID-19 and the pandemic continuing.

Our results were inconsistent with the recent study in Pakistan that showed that approximately 366 (77%) of students had negative perceptions of online learning. The reason for this is that the students and teachers in the previous study had to become acquainted with e-learning [14]. Unsurprisingly, our survey also showed that the students found that this new way of learning affected their behavior, ways of learning and learning schedules, but they still accepted online learning. In Taiwan, the e-learning process has been promoted by the Taiwan government since 1980 [16]. Hence, students in Taiwan are more likely to be adapted to online courses. Approximately 371 (80%) of the students felt that virtual learning had the same or better learning efficacy than on-site learning. Previous evidence also showed that there was no significant difference in student performance between online and on-site learners overall [17]. E-learning should be promoted and supported. Intelligent technology should be considered for the learning process of dental education during the pandemic [18].

Our results also showed that the surveyed students were confident that general dental lecture courses could be changed to online courses, but they did not feel this way about dental professional courses with a laboratory format. Therefore, for laboratory courses, we may consider blended learning or virtual reality education. Blended learning is a mix of traditional face-to-face learning and simultaneous or asynchronous e-learning. Because of its synthesized characteristics, this approach has been presented as a promising alternative approach for health education [19]. Its efficacy has recently been shown [20]. Furthermore, this approach was reported to improve the clinical practice of students who majored in health [21, 22]. Virtual reality technology can also be used for dental education and training, which allows students a safe environment before they have physical interaction with real patients. However, the efficacy of the use of virtual reality to teach operative skills in undergraduate students is still debatable [23]. More evidence supporting the efficacy of virtual reality dental education is needed.

SARS CoV-2 is a rapidly spread and deathly virus; however, the surveyed students were only moderately worried about being infected by the virus and the virus continuing to spreading. Dentists and dental practitioners need to avoid unnecessary contact, wear personal protective equipment while examining and working on patients, and follow universal measures to control the infection [24]. Fortunately, Taiwan has still not faced a crisis due to the pandemic. However, in the comparison of the responses of dental and non-dental students, dental students were more worried than non-dental students. This finding communicates a very important message to dental schools that they must ensure the safety of dental students.

Interestingly, dental students were more worried about financial pressure than non-dental students. The salary of dentists ranks in the top three in Taiwan (NT$81,478/month) [25]. The tuition fees for undergraduate programs in the School of Dentistry rank the second highest in the surveyed university (NT$130,820) and are double those of other undergraduate programs (NT$56,110). Students’ worries about financial pressure seemed to be unaffected by the high tuition fees themselves since the Ministry of Education stated that universities in Taiwan would not increase tuition fees. The pandemic has resulted in not only health impacts but also economic impacts in many countries, including Taiwan [26]. These economic impacts could affect parents who are responsible for their children’s educational fees.

Even though, the attitude toward online course are vary by countries [14, 27], we believe that there will be more demand on virtual learning in the future in dental education, for our result show a good attitude of dental students toward the virtual learning. Dental education in the countries that have enough resources (both of technology and human), for conducting online leaning should prepare for an E-learning in the future. However, there is a need for international cooperation to facilitate those countries that resource is limited to implement this platform.

In this study, we made use of an existing e-Learning online system. It has been implemented for ten years for the purpose of flexible learning mode, even before the pandemic of COVID-19. In this platform, faculties are encouraged, but not compulsory, to share tutorial notes, interactive quiz, homework assignment, and questionnaire and survey with students for each single course across all undergraduate and graduate programs. In this study, we took advantage of this platform to issue the anonymous questionnaire from students of College of Oral Medicine and had a satisfactory response rate of 70%.

Some limitations exist in our study. First, the generalizability of the results in our study is limited. We conducted a study at only one private medical university. Second, the surveyed targeted students did not include intern students; therefore, the results may be applicable only to campus students.

In conclusion, students in dental schools had positive attitudes toward online courses, and 366 (77%) of students agreed with changes to online learning. Therefore, changing to online learning seems to be feasible among students. However, more discussion about changing dental professional courses with a laboratory format to online courses is needed. Dental schools need to ensure the safety of dental students when they have an education during the COVID-19 pandemic.

## Supplementary Information

**Additional file 1.**

## Data Availability

The datasets used and/or analyzed during the current study are available from the corresponding author on reasonable request.

## Abbreviations

COVID-19: Coronavirus disease 2019
SARS CoV-2: Severe acute respiratory syndrome coronavirus 2
SAS: Statistical analysis system
HIV: Human immunodeficiency virus
HBV: Hepatitis-B virus
NT$: New Taiwan dollar
