# Supplementary material for: Dental education changed by COVID-19: Student’s perceptions and attitudes
Source: BMC Med Educ. 2021 Jul 3;21:364. doi: 10.1186/s12909-021-02806-5 (PMC8254053; doi:10.1186/s12909-021-02806-5)
Supplement: Supplementary file 1 — Additional file 1. [file 12909_2021_2806_MOESM1_ESM.docx]

**Questionnaire**

1. Department：______Degree: ______
2. Age：­_____
3. ( ) Sex：①Male②Female

**Part I**

1. ( ) **Are you agree with changing the on-site course to on-line course?**
   1. Very Disagree ②Disagree ③Neutral④Agree ⑤Very agree
2. ( ) **On-line courses change my learning schedule and behavior?**
   1. Very Disagree ②Disagree ③Neutral④Agree ⑤Very agree
3. ( ) **On-line courses change my way for learning?**
   1. Very Disagree ②Disagree ③Neutral④Agree ⑤Very agree
4. ( ) **I satisfy with the learning efficacy of on-line course?**
   1. Very Disagree ②Disagree ③Neutral④Agree ⑤Very agree
5. ( ) **The learning efficacy for on-line course is better than on-site course?**
   1. Very Disagree ②Disagree ③Neutral④Agree ⑤Very agree
6. ( ) **I think the other non-medicine professional course with lecture style can be changed to on-line course?**
   1. Very Disagree ②Disagree ③Neutral④Agree ⑤Very agree
7. ( ) **I think the dental professional course with lecture style can be changed to on-line course?**
   1. Very Disagree ②Disagree ③Neutral④Agree ⑤Very agree
8. ( ) **I think the dental professional course with laboratory style can be changed to on-line course?**
   1. Very Disagree ②Disagree ③Neutral④Agree ⑤Very agree
9. ( ) **I can tolerance how long for the learning style with on-line course?**
   1. 1 month ②3 months③6 months④9 months⑤12 months

**Part II**

1. **( ) Are you pessimistic about the development of COVID-19 virus？**
2. Very Disagree ②Disagree ③Neutral④Agree ⑤Very agree
3. **( ) Do you feel worried and sleepless when you think about the COVID-19 virus？**
4. Very Disagree ②Disagree ③Neutral④Agree ⑤Very agree
5. **( ) Would you actively collect and care about the news and development of the COVID-19 virus at home and abroad?**
6. Very Disagree ②Disagree ③Neutral④Agree ⑤Very agree
7. **( ) Would you actively collect the latest medical information about COVID-19 virus and in-depth knowledge?**
   1. Very Disagree ②Disagree ③Neutral④Agree ⑤Very agree
8. **( )** **Does the COVID-19 virus affect your original plan about choosing the career in the future?**
   1. Very Disagree ②Disagree ③Neutral④Agree ⑤Very agree
9. **( ) Does the COVID-19 virus change your social mode?**
   1. Very Disagree ②Disagree ③Neutral④Agree ⑤Very agree
10. **( ) Does the COVID-19 virus change your way of relaxing**
    1. Very Disagree ②Disagree ③Neutral④Agree ⑤Very agree
11. ( ) **Does the COVID-19 virus change your personal hygiene?**
    1. Very Disagree ②Disagree ③Neutral④Agree ⑤Very agree
12. ( ) **Does the COVID-19 virus change your daily schedule and habits?**
    1. Very Disagree ②Disagree ③Neutral④Agree ⑤Very agree
13. ( ) **Does the COVID-19 virus change your learning mode?**
    1. Very Disagree ②Disagree ③Neutral④Agree ⑤Very agree
14. **( ) Are you worried about being infected with the COVID-19 virus?**
    1. Very Disagree ②Disagree ③Neutral④Agree ⑤Very agree
15. **(　　　)** **Are you worried that the COVID-19 virus continue?**
    1. Very Disagree ②Disagree ③Neutral④Agree ⑤Very agree
16. **( ) Are you worried that the epidemic affect learning?**
    1. Very Disagree ②Disagree ③Neutral④Agree ⑤Very agree
17. **（ ）Are you worried that the COVID-19 virus affect the finance pressure for school study?**
    1. Very Disagree ②Disagree ③Neutral④Agree ⑤Very agree
18. **( ) Is your learning method changed by the current online learning mode ?**
    1. Very Disagree ②Disagree ③Neutral④Agree ⑤Very agree
19. **( ) How satisfied are you with the self-learning outcome of the current online learning model?**
    1. Very Disagree ②Disagree ③Neutral④Agree ⑤Very agree
20. **( ) Compared with physical course, do you think the current online learning is more effective?**
    1. Very Disagree ②Disagree ③Neutral④Agree ⑤Very agree
